# Release from incarceration, relapse to opioid use and the potential for buprenorphine maintenance treatment: a qualitative study of the perceptions of former inmates with opioid use disorder

**DOI:** 10.1186/s13722-014-0023-0

**Published:** 2015-01-16

**Authors:** Aaron D Fox, Jeronimo Maradiaga, Linda Weiss, Jennifer Sanchez, Joanna L Starrels, Chinazo O Cunningham

**Affiliations:** Albert Einstein College of Medicine, Bronx, NY 10461 USA; Montefiore Medical Center, Bronx, NY 10467 USA; New York Academy of Medicine, New York, NY 10029 USA

**Keywords:** Opioid use disorder, Buprenorphine maintenance treatment, Incarceration, Access to care

## Abstract

**Background:**

The United States has the highest rate of incarceration in the world (937 per 100,000 adults). Approximately one-third of heroin users pass through correctional facilities annually. Few receive medication assisted treatment (MAT; either methadone or buprenorphine) for opioid use disorder during incarceration, and nearly three-quarters relapse to heroin use within 3 months of release. This qualitative study investigated barriers to and facilitators of buprenorphine maintenance treatment (BMT) following release from incarceration (“re-entry”).

**Methods:**

We conducted 21 semistructured interviews of former inmates with opioid use disorder recruited from addiction treatment settings. Interviews were audio-recorded, transcribed, and analyzed using a grounded theory approach. Themes that emerged upon iterative readings of transcripts were discussed by the research team.

**Results:**

Participants reported adverse re-entry conditions, including persistent exposure to drug use and stressful life events, which were perceived to contribute to opioid relapse and affected addiction treatment decisions during re-entry. Themes that emerged relating to BMT included: 1) reliance on willpower; 2) fear of dependency on medications; 3) variable exposure to buprenorphine; and 4) acceptability of BMT following relapse. Willpower was perceived to be more important for recovery than medications. Many participants experienced painful withdrawal from methadone during incarceration and were fearful that using MAT would lead to opioid tolerance and painful withdrawal again in the future. Participants reported both positive and negative experiences taking illicit buprenorphine, which affected interest in BMT. Overall, BMT was perceived to be a good treatment option for opioid use disorder that could reduce the risk of re-incarceration.

**Conclusions:**

BMT was perceived to be acceptable, but former inmates with opioid use disorder may be reluctant to utilize BMT upon re-entry. Factors limiting utilization of BMT could be mitigated though policy change or interventions. Policies of the criminal justice system (e.g., forced detoxification) may be dissuading former inmates from utilizing effective treatments for opioid use disorder. Interventions that improve education and access to BMT for former inmates with opioid use disorder could facilitate entrance into treatment. Both policy changes and interventions are urgently needed to reduce the negative consequences of opioid relapse following re-entry.

## Introduction

The United States has the highest rate of incarceration in the world (937 per 100,000 adults). At the end of 2011, 7 million Americans were under correctional supervision, including 2.2 million held in jail or prison [1]. Of those incarcerated, nearly two-thirds (1.5 million) have substance use disorders, including up to one-quarter with opioid use disorder [2-4]. It has been estimated that one-third of heroin users pass through correctional facilities annually [5]. Few inmates with opioid use disorder receive addiction treatment during incarceration, and rates of relapse and opioid overdose-related deaths (109 deaths per 100,000 person years, or 15 percent of all deaths among former inmates) are tragically high following release [6-9].

Medication assisted treatment (MAT), using methadone or buprenorphine, are the most effective treatments for opioid use disorder, with decades of research demonstrating reductions in opioid abuse, HIV risk behaviors, and criminal recidivism [10,11]. However, of the 28 state prison systems that offer any methadone treatment, over half limit treatment to pregnant women or individuals with chronic pain, and only seven state prison systems offer any buprenorphine treatment [12-14]. Consequently, for some, opioid abuse continues during incarceration [15,16]. Even when inmates maintain abstinence from opioids during incarceration, because opioid use disorder is a chronic relapsing illness, they are at high risk for opioid relapse following release [17,18]. Randomized controlled trials and observational studies have demonstrated that starting methadone or buprenorphine prior to release improves entrance into and retention within addictiontreatment and reduces opioid abuse following release [7,19-23], but data regarding the impact of pre-release MAT on recidivism has been conflicting [24,25]. Nonetheless, these practices have not become standard of care.

Relapse to opioid use following release from incarceration (“re-entry”) is extremely common, which makes the period of community re-entry following release critical to engage former inmates with opioid use disorder in treatment. Nearly three-quarters of prisoners with opioid use disorder relapse within 3 month of release, and with simple referral to MAT upon release, as few as 8 percent enter treatment [21]. Qualitative research focused on community re-entry has highlighted factors that contribute to relapse, including poor social support, inadequate economic resources, and widespread exposure to drug use, but barriers to addiction treatment were not investigated [26,27]. Barriers to medical care during re-entry include competing priorities, lack of knowledge about services, long waiting times, and costs of treatment, but there may be additional factors specific to addiction treatment and MAT that prevent former inmates from entering treatment [28]. Understanding how the experience of community re-entry affects former inmates’ attitudes toward and access to MAT could aid in development of interventions that encourage treatment, thereby preventing relapse, overdose, and re-incarceration.

Buprenorphine maintenance treatment (BMT) is an increasingly utilized, efficacious, and safe treatment for opioid use disorder, and it has several features that may enhance acceptability [29-33]. Private physicians in office-based settings can prescribe BMT, which may reduce stigma; side effects may be more tolerable than methadone; and patients may self-administer BMT, as opposed to methadone maintenance treatment (MMT) programs, where nurses administer and directly observe most dosing [34-36]. Qualitative data suggest that former inmates may prefer BMT to MMT [36]. Therefore, BMT has promise for reducing the consequences of opioid relapse during community re-entry, but it has yet to be fully realized.

To better understand why former inmates would or would not initiate MAT in general, and BMT more specifically, we conducted a qualitative study of the barriers to and facilitators of BMT following re-entry. Findings from this study will inform an intervention to facilitate entrance into BMT following re-entry and can be used to develop policy regarding MAT and incarceration.

## Methods

We conducted semistructured interviews with 21 former inmates with opioid use disorder between November 2012 and December 2013. The study was approved by the Institutional Review Board of the Albert Einstein College of Medicine.

### Participants

Inclusion criteria were: 1) incarceration (≥1 day in jail or prison) in the previous 5 years; 2) opioid use disorder; 3) 18 years of age or older; and 4) fluent in English or Spanish. Sampling was by convenience, but we targeted recruitment to include participants with variability regarding experiences taking buprenorphine (BMT, illicit buprenorphine only, and no buprenorphine) and length of time since re-entry. Primary care providers at a federally qualified health center (FQHC) that houses an office-based BMT program [37] and a re-entry program for former inmates [38] referred patients who met inclusion criteria. Drug counselors at an outpatient substance abuse treatment program that provides nonpharmacologic addiction treatment to former inmates (often as part of parole requirements) also referred clients to study. This ensured inclusion of those who sought addiction treatment by choice and those who were mandated to treatment.

### Interviews

After obtaining informed consent, face-to-face interviews lasting approximately one hour were conducted in a private room at the FQHC or substance abuse treatment program. All interviews were audio-taped and professionally transcribed (and translated to English when necessary), and participants were compensated with $20 and a $5 transit pass. An interview guide was developed to elicit participants’ experiences with opioid use disorder, incarceration, and community re-entry; attitudes toward MAT; and beliefs about buprenorphine. Interview questions were based on Social Cognitive Theory (SCT), which models a pathway of behavior change that has frequently been applied to addiction [39]. According to SCT, self-efficacy, outcome expectations, goals, and structural barriers/facilitators are all important domains relating to behavior change; therefore, interview questions addressed these domains in relation to why or why not participants engaged in MAT in general, and BMT more specifically, during community re-entry.

### Analysis

We analyzed the data in an iterative process using a Grounded Theory approach. Three investigators (ADF, JM, JS) developed a coding scheme to categorize common themes that emerged upon iterative readings of the first five transcripts. Several codes overlapped with domains of SCT (e.g., goals for addiction treatment following release), and others related to common experiences (e.g., returning to housing that exposed participants to substance use). This coding list was then applied to all 21 transcripts with two investigators independently coding each one. Transcripts were then discussed by the entire research team, and discrepancies in coding or revisions to the coding list were resolved by consensus. Agreed-upon codes were entered into N-Vivo software, so that content from all transcripts could be sorted and extracted by code. The research team then retrieved and discussed content from all transcripts by code to further understand and refine the common themes.

## Results

Participants were mostly male, Hispanic or African American, English-speaking, and the median age was 49 (see Table 1). They had used heroin for a median of 24 years (interquartile range [IQR]: 15–30) in their lifetimes, and six reported using heroin in the previous 30 days. Nearly all had a history of injection drug use, MMT, and had received abstinence-based addiction treatment during incarceration. They had been incarcerated for a median of 16 years (IQR: 5.5–26) as adults, and release from prison or jail was a median of 7.5 months (range: 10 days–4 years) prior to the interview. Overall, 12 participants had taken buprenorphine, including eight who had ever received BMT, six who received BMT at the time of the interview, and four who had tried illicit buprenorphine but had never received BMT. Experience with relapse varied, with more than two-thirds relapsing to opioid use during community re-entry, and only half of these participants starting MAT.

**Table 1:** **Sociodemographic characteristics of former inmates with opioid use disorder**

**Characteristic**	**N (%)**
Age, median years (IQR)	49 (46–52)
Male	17 (81)
Race/Ethnicity	
Hispanic	13 (62)
Non-Hispanic Black	8 (38)
English-speaking	20 (95)
Medicaid	19 (90)
High school diploma or equivalency	11 (52)
Ever injected drugs	15 (71)
Current substance use^a^	
Heroin	6 (29)
Other opioid analgesics	2 (10)
Cocaine	3 (14)
Lifetime substance use^b^	
Heroin	21 (100)
Other opioid analgesics	9 (43)
Cocaine	17 (81)
Treatment history	
MMT	13 (62)
Nonpharmacologic treatment	20 (95)
BMT	8 (38)
Any opioid addiction treatment	21 (100)

^a^Within the previous 30 days.

^b^Regular use within lifetime.

During community re-entry, most participants experienced adverse re-entry conditions, which included structural or psychosocial challenges that affected decisions about addiction treatment, and often which led to opioid relapse. Participants’ experiences regarding post-release opioid addiction treatment varied greatly, with a few starting BMT immediately following release and others refusing any type of MAT. Decisions regarding MAT in general and BMT specifically fell within four broad themes: 1) reliance on willpower; 2) fear of dependency on medications; 3) variable exposure to buprenorphine; and 4) acceptability of BMT following relapse. Below, we describe the adverse re-entry conditions and then elaborate on each of these themes, including how they were barriers to or facilitators of BMT.

### Adverse re-entry conditions

Decisions regarding addiction treatment during community re-entry were made within the context of stressful life events and living conditions that made long-term abstinence difficult. Both structural factors, like exposure to drug use in halfway houses or shelters, and psychosocial factors, such as isolation and hopelessness, were accentuated during re-entry and were perceived to contribute to relapse. A participant who relapsed to heroin use during re-entry and subsequently started BMT described his housing conditions:

*I lived in the halfway house…and a lot of people get high, people selling drugs, you go in the bathroom and find empty heroin bags, people leave their works in there. (Participant 1)*

Other participants described the stress of living with family members and needing to contribute to rent, despite not having employment or a source of income. Additionally, relationships with family members were often strained by the participant’s history of addiction and repairing these relationships was stressful. One participant described the impact of substance use and incarceration on her family:

*The reason why I couldn’t stop [using drugs] is because you lose your custody of your kids, your family, they don’t trust you and you’re just like an outcast, and they’re waiting and expecting you to just relapse and it hurts you. It hurts you emotionally. (13)*

Participants also commonly described the material things or relationships that they had lost due to incarceration and how difficult it was to start over during community re-entry; this often led to hopelessness. One participant who had maintained abstinence following his most recent incarceration reflected on the challenges of finding meaning in post-incarceration life and avoiding relapse:

*Could you imagine – a person comes home and they have nothing. They’ve lost their family. They have no prospects for a job. They have almost literally nothing to live for. What is going to stop that person from saying well, let me just get high? I have nothing else. (5)*

A few participants reported having a positive social support system, including spouses or re-entry programs, but more commonly, participants were socially isolated and unprepared for the challenges of community re-entry.

The following themes contributed to decisions about MAT in general, and BMT more specifically:

#### Reliance on willpower

Participants expressed that not using opioids was a personal decision and emphasized that individual traits like willpower and readiness to change were more important than participation in addiction treatment. This reliance on willpower could be a barrier to MAT; it also led to internal conflict if participants relapsed. Nearly all participants commented that accepting personal responsibility for addiction was an essential first step in not using opioids. One participant who was abstinent at the time of the interview explained why he chose not to start BMT during re-entry:

*Programs, doctors, drugs, whatever, they could only do so much. The person has to bear some kind of responsibility in this whole equation. I have to take responsibility for my own life. (5)*

Another participant who was offered BMT by his physician during community re-entry, but had refused BMT, also emphasized personal responsibility:

*The first things is the desire to overcome [addiction]. If you want to overcome it, that’s number one. If you’re not interested, you’re not going to go anywhere. No matter how much they try to help you, they can’t go inside your head and obligate you. It has to be you. (11)*

However, accepting personal responsibility for addiction seemed to be necessary but not sufficient to prevent relapse. Participants described prior episodes of community re-entry in which they had felt ready to stop using opioids, yet still experienced relapse. One participant recognized that he had been in similar situations previously and had relapsed:

*Sometimes I thought – I tried to deceive myself. This time I’m going to do things right. I didn’t last a month before I was back selling and using drugs. (11)*

Another participant who had felt ready to stop using opioids at release, but still relapsed to heroin use, described the internal conflict. Following relapse, this participant started BMT and was able to maintain abstinence, but he reflected on his struggles when relying on willpower alone:

*Maybe I just didn’t have no willpower, and just had to use. But I really didn’t. I was really trying to contemplate a scenario where I wouldn’t be using heroin. You know what I’m saying? And it didn’t work out like that, you know. (1)*

Regardless of past experiences with relapse, many participants still believed that if they had sufficient willpower, they would be able to stop using opioids. Reasons for wanting to stop included parental responsibilities, failing health, fear of going back to prison, or simply “being tired”. Overall, there was a sense of confidence at the time of the interviews that they would be able to stop using opioids, even among participants who had already relapsed since re-entry. One participant who had used oxycodone and heroin in the 30 days prior to the interview, but who was not interested in starting BMT, reported:

*I think drugs have outran their course in me…they have really made me see the error of me, though. At times, I rely on the high to not feel something that I’m going through at that particular moment. But, I’ve grown to understand that whatever I go through and if I’m [thinking] a drug is going to erase it, it’s not. (19)*

#### Fear of dependency

When participants did receive addiction treatment, there was often a preference for medication-free approaches due to the fear of becoming dependent on opioids. Though a few participants started BMT or MMT immediately following release, for most participants, their goal was to be free of opioids (“clean”), which included medications. There were two main reasons for the medication-free preference: dependence on medications was perceived as a step backward in recovery, and it was a setup for painful withdrawal in the future.

##### A step backward

Though a few participants managed to use enough illicit opioids during incarceration to maintain opioid tolerance (or “a habit”) for the most part, participants drastically reduced their opioid use while incarcerated and perceived themselves to be “clean” at release. A participant who had experience with both MMT and BMT described how using MAT at release was counter-intuitive for him:

*I’ve been incarcerated for 8 years…as soon as I’m going home I’m going to a methadone program? Why do you need to go to a methadone program when you’ve been 8 years clean? (6)*

Another participant who later entered MMT commented on his mentality at release, “*I don’t have no drugs in me. I don’t need [buprenorphine]…I’m clean”.(20)* For this participant, and others who utilized MAT, the dependency on medication seemed like a failure, because they still maintained a goal of being opioid-free. This participant spoke about “trading one drug for another” and the psychological effect of MAT, “*Right now I’m in the methadone program. I know that’s legal and everything, but I don’t feel clean. The same thing for [buprenorphine]”*. *(20)*

##### Setup for withdrawal

Participants’ experiences with withdrawal from methadone while incarcerated also contributed to the fear of dependency. For participants who were receiving MMT when they were incarcerated, all were involuntarily and rapidly detoxified from methadone and experienced prolonged withdrawal symptoms. The withdrawal symptoms were severe, persisted for months, and traumatized participants. One participant described the severity of the withdrawal symptoms:

*Since I was in the meth program, they detoxed me and it was really cruel. I was going crazy. I wanted to hang myself and I couldn’t deal with it. Honestly, when I finished, I was in so much pain that I had to go to the psychiatrist so I could try to get some type of pill. (16)*

The trauma of these experiences often resulted in an aversion to methadone that could eliminate MMT as an acceptable treatment option, “*I would never take methadone again. Hell, no. I suffer[ed]”*. *(6)* The aversion to methadone did not seem to convey negative attitudes toward buprenorphine, and for a few participants, the painful withdrawal during incarceration led them to try BMT as an alternative. There were no participants who were receiving BMT at the time of incarceration, so any aversion to BMT was due to prior adverse reactions to buprenorphine, not because of their experience with withdrawal from buprenorphine.

#### Variable exposure to buprenorphine

During incarceration, BMT was not prescribed and was not part of participants’ addiction education; however, most had either taken or heard about illicit buprenorphine before or during incarceration. This haphazard exposure to buprenorphine made participants’ experiences variable, which affected attitudes toward BMT. Several participants reported negative experiences taking buprenorphine, but this was in the context of illicit buprenorphine use, without the supervision of a physician. One participant who had relapsed to opioid use following release, but who was not interested in BMT, reported a prior negative experience taking illicit buprenorphine:

*The dude just gave [buprenorphine] to me and didn’t tell me I had to be careful and I took the whole thing. All of a sudden I lost control of everything. My bowel movement, my urine, everything. My equilibrium was totally thrown off track. And when I got home I fell out and woke up in the hospital. (14)*

Alternatively, when illicit buprenorphine was used effectively to self-treat withdrawal symptoms during incarceration or when BMT was provided by a physician during re-entry, participants reported more positive attitudes toward BMT. One participant had taken illicit buprenorphine during a short jail stay to self-treat withdrawal symptoms and then started BMT during re-entry:

*That’s how I found out about the [buprenorphine] in jail…I got so sick in there — sweating, nightmares, you couldn’t even go to sleep right…I mean I never ever heard about [buprenorphine] and [another inmate] gave me a little piece and I put it in my tongue and they calmed me down. (2)*

#### Acceptability of BMT following relapse

Despite the reliance on willpower and fear of dependency, for the majority of participants, BMT was perceived as a good way to prevent re-incarceration and commonly was seen as a better option than MMT. In response to questions about the ideal treatment approach to prevent relapse during re-entry, participants recommended more widespread use of BMT through improved education, access to physicians, or specific programs that could be implemented within correctional facilities. One participant who had relapsed during re-entry and then started BMT believed that BMT should routinely be offered during incarceration:

*The average person, they would be discharged from jail or prison, [they’re] scared okay. And 95 percent of the time drugs [are] what got them in prison or jail. And, a lot of them don’t want to go back to that. But if you had like these pre-release programs and let them know you have an alternative, you don’t have to go on the methadone program, you can have [buprenorphine] and then follow suit so that you won’t get locked up. (9)*

Even among participants who had never tried buprenorphine, there was recognition that BMT could prevent re-incarceration and would be a viable option if they needed it. Several participants had spouses or family members who received BMT. One participant who chose not to receive MAT still had a positive impression of BMT:

*I heard that it stops the craving. I see my brother stable now. He’s not going out there searching for a bag of dope or trying to sell something. He’s calm. He’s got a good job. (12)*

Overall, BMT was perceived to be acceptable, but often participants chose to wait to enter treatment until they had a “dirty urine” (i.e., following relapse to opioid use). One participant who had previously received BMT but elected for medication-free treatment following his most recent re-entry described BMT as an acceptable “Plan B” (8) if group treatment did not work for him. For other participants, it took relapse before they sought treatment, but ultimately, they were able to access BMT and they acknowledged that the medication helped them avoid re-incarceration:

*Soon after I started back using [drugs], these two kids in the halfway house told me about [buprenorphine]. I already had a doctor so I asked him about it and we discussed it. And I was concerned about being on parole and taking [buprenorphine], and he was like, “Trust me. Your parole officer would prefer you to be on [buprenorphine] than heroin”. [Now], I don’t have cravings for heroin, and if I had cravings for heroin, I’d be back into using heroin again. And that would, you know, automatically turn back to a life of crime. And that will definitely get you back to prison. (1)*

## Discussion

In this qualitative study on perceptions of opioid addiction treatment during community re-entry, participants universally reported adverse re-entry conditions, and we identified several important themes relating to decisions about BMT. Reliance on willpower and fear of dependency often led participants to favor medication-free treatment. However, following relapse, BMT was seen as an acceptable treatment option that had the potential to prevent re-incarceration, especially among participants who had positive experiences or impressions of buprenorphine.

Our study expands the literature on post-incarceration relapse to substance use by offering insights into why former inmates may not seek MAT. Two other recent qualitative studies described the re-entry experiences of substance users and have similarly documented the adverse re-entry conditions, including exposure to drug use and ubiquitous psychosocial distress [26,27]. Several studies have also demonstrated gaps in knowledge about BMT among more general populations of opioid users [40-42]. Our study builds upon these studies by demonstrating how attitudinal barriers, such as fear of dependency and the potential over-reliance on willpower, may also affect decisions about BMT during community re-entry. While correctional systems or courts often favor abstinence-based approaches to opioid addiction treatment [12], our participants’ preference for medication-free approaches to treatment was surprising. This emerging literature is far from complete, and a better understanding of treatment seeking and treatment fears will be critical for designing policy and interventions to reduce relapse during community re-entry.

Our findings reiterate that opioid use disorder is a chronic disease that is not cured by the forced abstinence of incarceration. Our participants desired to be opioid-free but were unprepared to face the adverse re-entry conditions. During re-entry, most relapsed and either started MAT or continued to use opioids. It is possible that some participants will achieve sustained abstinence, but it is unlikely that reliance on willpower alone will be sufficient for most. Our study highlights the need for additional opioid addiction treatment options, including MAT and longitudinal aftercare, for most former inmates with opioid use disorder during community re-entry. With the high rate of opioid overdose death immediately following release [8] and the data demonstrating that pre-release MAT increases utilization of addiction treatment during re-entry [7,19-23], we question the wisdom of releasing inmates who have long histories of opioid use disorder without at least offering MAT. Our data suggest that while some will be reluctant to start MAT, with better education and linkage to treatment, others would find BMT acceptable, and starting treatment could avert relapse and overdose-related deaths.

One theme, the fear of dependency, is of particular interest because it could limit utilization of effective treatments. The stigma of MAT or “trading one addiction for another” is well known [43], but correctional policies that require rapid detoxification from MAT may also be exacerbating this fear of dependency. After repeated incarcerations, opioid users’ reluctance to receive MAT may reflect an acceptance that future incarceration is probable, as well as their desire to avoid the painful withdrawal that accompanies involuntary withdrawal of MAT during incarceration. Our findings are consistent with one past study that documented the experience of opioid withdrawal during incarceration [44]. There are two major implications. First, policies of the criminal justice system, including involuntary withdrawal of MAT during incarceration and re-incarceration for minor parole violations, may be causing physically painful disruptions in treatment that dissuade individuals with opioid use disorder from continuing the most effective forms of treatment. Second, for participants who are fearful of developing tolerance, opioid-antagonist treatments, such as naltrexone, may be a better option. As an opioid antagonist, naltrexone does not cause withdrawal upon cessation, and there is good evidence that long-acting formulations can reduce opioid abuse, improve retention in treatment, and may prevent opioid overdose deaths [45-48]. There is also pilot data supporting the feasibility of using injectable naltrexone in criminal justice settings in the United States [49]. Our participants mostly described the traumatic withdrawal from methadone during incarceration, so the impact of withdrawal from buprenorphine is still unknown but warrants additional investigation.

There are other implications of our findings. Interventions should address inmates’ incomplete knowledge about BMT and facilitate their linkage to MAT prior to relapse. Participants with negative experiences taking illicit buprenorphine (e.g., “it almost killed me”) may have used buprenorphine concomitantly with other opioids and therefore precipitated withdrawal. Buprenorphine education and proper induction procedures could limit these negative experiences. A number of participants who were unaware of buprenorphine at release were eventually able to initiate BMT following the suggestion of friends, counselors, physicians, or even parole officers. This suggests that routinely facilitating entrance into treatment during community re-entry could prevent relapse (or at least, shorten the time between relapse and treatment entry). Interventions to address the adverse re-entry conditions faced by all our participants, including lack of housing and employment opportunities, will be more difficult to implement, but these efforts will also be necessary to prevent relapse and support successful re-entry.

Our study has limitations. We recruited a convenience sample from addiction treatment settings, so attitudes of out-of-treatment former inmates with opioid use disorder may not be represented; however, this targeted recruitment allowed us to understand barriers for those who are ready to stop using opioids and would be amenable to treatment interventions. Our sample was also mostly middle-aged former prisoners with long histories of opioid use disorder, so these findings may not be generalizable to younger opioid users with less incarceration experience, who may have different attitudes toward entering opioid addiction treatment. All interviews were conducted in New York, which offers Medicaid coverage for BMT, so access to and knowledge of BMT may be greater than in other geographic areas. Methadone detoxification upon incarceration is also readily available in New York City jails; therefore, the experience of opioid withdrawal upon incarceration may be worse in other geographic areas. Finally, because many of the participants were on parole, they may have been unwilling to fully disclose relapse; however, we attempted to understand treatment-seeking decisions, not evaluate the effectiveness of treatment or the incidence of relapse.

## Conclusions

The cycle of incarceration, release, relapse, recidivism, and re-incarceration has been a problem for individuals with opioid use disorder for decades. Recently, there have been efforts to break the cycle by providing enhanced re-entry services or offering addiction treatment as an alternative to incarceration. These efforts are important, but may be insufficient if MAT, including BMT, is not offered as part of the treatment approach. No one intervention alone will break the seemingly intractable cycle of incarceration, but BMT appears to be acceptable, and with targeted interventions, holds great promise for engaging former inmates in opioid addiction treatment.
